# Horn bud size of dairy-bred and suckler-bred calves at time of disbudding

**DOI:** 10.1186/s13620-021-00196-0

**Published:** 2021-06-16

**Authors:** Gabriela A. Marquette, Mark McGee, Andrew D. Fisher, Kelly Stanger, Anastasio Argüello, Bernadette Earley

**Affiliations:** 1 Teagasc, Animal & Grassland Research and Innovation Centre, Grange, Dunsany, Co. Meath, C15 PW93 Ireland; 2 Faculty of Veterinary and Agricultural Sciences, University of Melbourne, Melbourne, Australia

**Keywords:** Calf breed, Age, Sex, Horn bud size, Cautery disbudding

## Abstract

**Background:**

Hot-iron disbudding is a common management procedure to prevent horn growth in calves. The study objective was to examine effect of age, breed and sex on horn bud size of dairy-bred and suckler-bred calves at time of disbudding.

**Results:**

The left and right horn bud size (diameter and height in mm) of 279 calves, including dairy-bred Holstein-Friesian (Male (M) = 88) and 191 suckler-bred (86 Charolais, CH; (M = 39, Female (F) = 47), 67 Limousin, LM; (M = 32, F = 35) and 38 Simmental, SI; (M = 22, F = 16) sired)) was measured using a digital calliper at time of disbudding. Calves were retrospectively assigned to two age categories at time of disbudding: 1), 14 to 28 days (d) old and 2), 29 to 60 d old. Holstein-Friesian M calves had a greater horn bud diameter (16.97 *v.*14.45 mm) and height (7.79 *v.* 5.00 mm) compared to suckler-bred M calves (*P <* 0.01), with no difference (*P >* 0.05) among the suckler-bred calves. Suckler-bred M calves had a greater horn bud diameter (14.46 vs 13.29 mm) and height (5.01 vs 3.88 mm) compared to suckler-bred F calves (*P <* 0.05).

The slopes of the lines of best fit show that horn bud diameter and height increased with age (*P <* 0.05) for HF, SI male and CH female calves while there was no relationship with age (*P >* 0.05) for CH and LM male calves, or for SI and LM female calves. Linear regression of age with diameter and with height for each breed and sex showed high variability in the data as indicated by R-squared values ranging from 0.003–0.41 indicating that in the case of the diameter and the height, the weight of the fitting effect was poor.

**Conclusions:**

Calf age is not a good predictor of horn bud size and recommendations for the disbudding of calves should be based on horn bud size and not on age. The implications of these findings are that calves should be disbudded while horn development is still at the bud stage and when the bud is large enough to be easily palpable/visible, but not so large that disbudding could lead to severe tissue trauma.

## Background

Disbudding and dehorning are husbandry management procedures, which as a principle are deemed undesirable by society, but permitted in law because there are perceived benefits to human and animal safety. The primary reasons stated for removal of horns or horn buds is to make the handling of cattle easier and to reduce the risk of injuries (in cattle, other animals and human handlers) associated with horned cattle [1] Disbudding involves the destruction of the cells of the horn bud [2, 3] and is defined as the removal of horns in calves up to 2 months (mo) of age [1]. Dehorning refers to the removal of the horn after attachment of the horn bud to the skull, occurring at approximately 2 mo of age [4–6]. Farmers as well as veterinarians, in many countries, consider removal of the horn buds a painful procedure for calves [7–12]. Disbudding and dehorning procedures are therefore regulated by animal welfare laws. Within the European Union (EU), the disbudding procedure is regulated by European Council Directive 98/58/EC based on the Recommendation Concerning Cattle [13]. In addition, cautery disbudding is recommended by the European Food Safety Authority [14], and is the only method allowed in Ireland under S.I. One hundred twenty-seven of the Animal Health and Welfare [15], which permits disbudding of calves up to 4 weeks of age by thermal cauterisation. Cornual nerve block with local anaesthetic is currently the accepted technique to anaesthetize the horn bud prior to its removal [16].

Under Irish legislation, calves can be disbudded under 2 weeks of age without use of local anaesthesia or analgesia. Administration of a local anaesthetic as a prescription only medicine (POM) by a non-veterinarian stockperson is permitted under Irish legislation for the disbudding of calves from 2 weeks to 4 weeks of age. For calves older than 4 weeks, administration of a local anaesthetic prior to disbudding under veterinary supervision, is mandatory.

A review of the literature indicates that there are differences among countries in the use of hot-iron cautery disbudding. For example, in the Czech Republic, 69.4% of farmers use the hot-iron cautery disbudding procedure [17], which is similar to the 69.1% reported in the United States [18] and is lower than that reported in Canada (88.7% [19]), Brazil (95% [20];), Italy (90.6% [6];), France (87%, [21]), 87.6% [22]) and EU member states (80.4% [1]). In Ireland, the age limits concerning the timing of the disbudding procedure for calves and the administration of local anaesthesia, regardless of breed or sex, are not based on empirical evidence, but rather on farmer opinion related to the physical development of horns [23]. Additionally, there are no recommendations in Ireland as to the provision of pain relief (analgesia) based on age. Research conducted by Dwane et al. [23] using focus groups reported that Irish beef farmers enrolled in the “Suckler Scheme 2008” [24] were reluctant to use local anaesthetic and opted to disbud suckler-bred calves younger than 2 weeks old to avoid using it, even when the horn bud was not palpable. While the ‘Disbudding’ measure may have been beneficial in reducing the incidence of dehorning of adult cattle [25], early disbudding could result in re-growth and having to repeat the procedure a second time in calves where it was not successful.

Despite the visibility of the horn bud being the characteristic normally adopted by farmers when selecting animals for disbudding, most recommendations are still based on the age of the calf. Research indicates that young calves may be equally or possibly more sensitive to pain than older calves [26], and raises doubts about the validity of age-related anaesthetic and analgesia guidelines. Information is lacking on; 1), the bud size (diameter and height) and age at disbudding and 2), on horn bud size (diameter and height) of contrasting breeds, and of calf sex and if the recommendation to disbud calves based on age is appropriate for all calf breeds.

Therefore, the objective of this study was to examine the effect of age, breed and sex on horn bud size of calves at time of disbudding. Our hypothesis was that the relationship between calf age and horn bud size would differ between breeds, and thus any recommendation for an optimal disbudding age may need to take breed into account, or instead be based on horn bud size itself rather than calf age.

## Materials and methods

### Animals

A total of 279 calves, including 88 dairy-bred Holstein-Friesian (Male (M)) and 191 suckler-bred (86 Charolais, CH; (M = 39, Female (F) = 47), 67 Limousin, LM; (M = 32, F = 35) and 38 Simmental, SI; (M = 22, F = 16)) sired calves) were used. The Holstein-Friesian M calves were purchased directly from farms in 2018 (*n* = 45) and 2019 (*n* = 43) and artificially reared at Teagasc, Grange, Co. Meath. The suckler herd at Teagasc was the source of the suckler bred calves. The cows, Limousin and Simmental crossbred cows were bred to artificial insemination (AI) to Charolais, (CH) Limousin (LI) and Simmental (SI) sires. The M and F suckler-bred calves were spring-born at Teagasc, Grange in 2018 (*n* = 88) and 2019 (*n* = 103). The HF calves were the progeny of HF sire with HF dam, there was no Jersey breed influence. All calves were retrospectively assigned to two age categories based on birth dates: (i) 14 to 28 d (ii), 29 to 60 d.

### Local anaesthesia and analgesia

Each calf was restrained in a disbudding crate. Cornual nerve blockade was administered by a trained farm technician by injecting 2 mL of local anaesthetic (LA) solution containing Procaine hydrochloride (50 mg/mL) with adrenaline (0.2 mg/mL) (Adrenacaine, Norbrook Laboratories Limited, UK). The site of LA injection was located by running a finger along the head from the lateral canthus of the eye to the horn bud on each side of the head. About 3 cm from the eye along this line, the needle was inserted through the skin under the bony ridge and the anaesthetic deposited. Each HF calf was also administered a subcutaneous injection 0.5 mg/kg containing 20 mg/mL of meloxicam (Metacam 20 mg/mL, Boehringer, Ingelheim, Boehringer, Germany). The suckler bred  beef calves were not administered metacam.

### Horn bud measurements

**Fig. 1:**
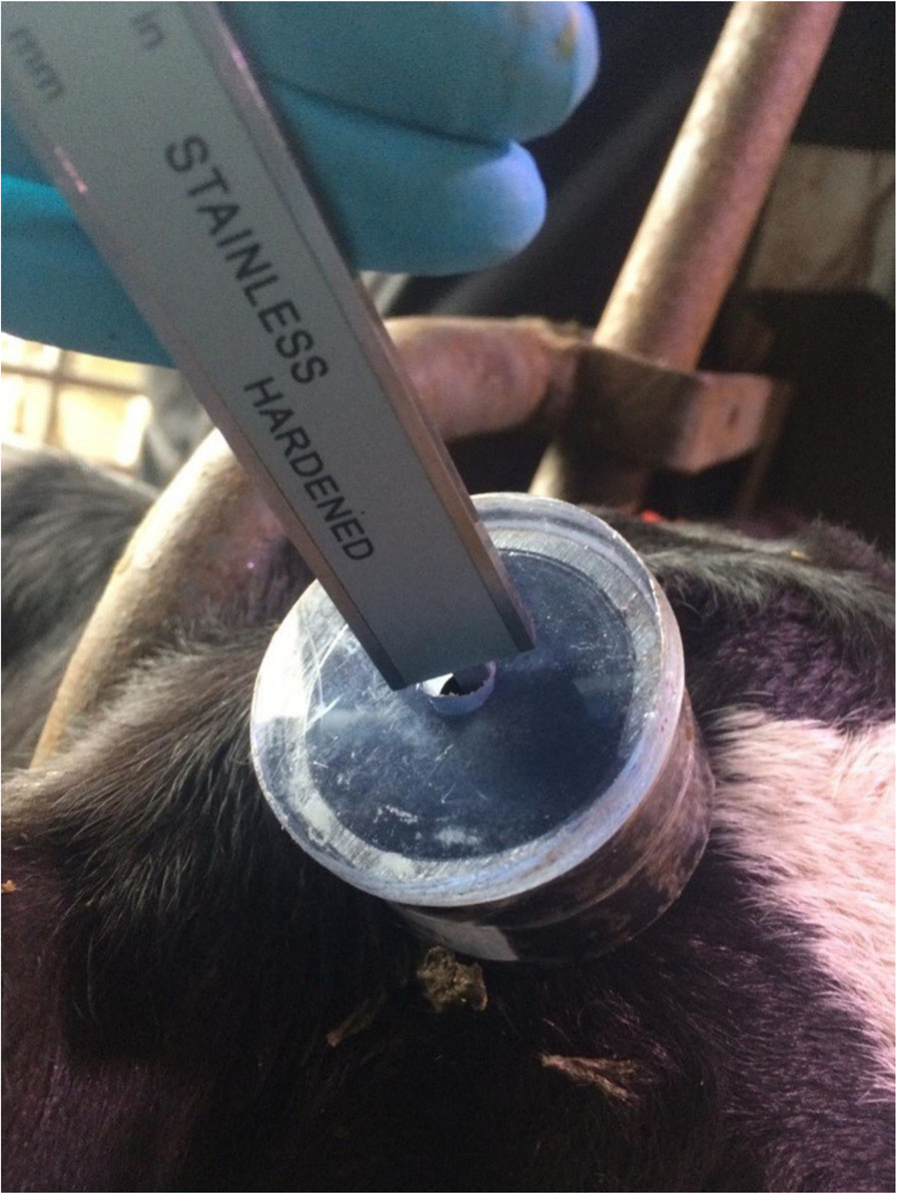
Methodology used to measure horn bud height showing the depth gauge of the calliper (model 49–923-150; Linear Tools, UK) positioned through the aperture of the cylinder (diameter 43 mm; height 30.85 mm) when placed over the horn bud

At the time of disbudding, 20 min after the application of a cornual nerve block using LA and analgesia, calves were moved individually to a disbudding crate and restrained. The hair surrounding each horn bud was clipped using a scissors and the horn bud exposed. The diameter (mm) and height (mm) of the left and right horn buds were measured using a digital calliper (model 49–923-150; Linear Tools, UK) and a clear plastic cylinder (diameter 43 mm; height 30.85 mm) which was placed overlying the horn bud prior to disbudding (Fig. 1). The horn bud height was determined using the depth gauge on the calipers as the difference between the depth measured through an aperture in the top of the cylinder to the tip of the horn bud, and the cylinder height. The diameter was determined by placing the outside jaws of the calipers parallel to the horn bud. The same individual carried out all of the horn bud measurements.

Following the horn bud measurements, each calf was disbudded using the farm’s standard management practice, using a hot-iron disbudder (model 17,460; KERBL, AgriDirect, Ireland) with a tip diameter of 15 mm. Silver aluminium spray (Henry Schein, Dublin, Ireland) was applied to the disbudded area following removal of each horn bud and the calves were returned to their home pens.

### Statistical analysis

Statistical analyses were conducted using SAS v.9.4 software [27]. The experimental unit was the animal. A descriptive analysis of the horn bud measurements collected on the right and the left side of each calf was performed in SAS. Since no differences were found between the left and right side horn bud measurements, the mean value for diameters and heights of each calf was used in the subsequent analysis. Data were checked for normality and homogeneity of variance by histograms, q–q plots, and formal statistical tests as part of the UNIVARIATE procedure of SAS. Data that were not normally distributed were transformed by raising the variable to the power of lambda. The appropriate lambda (λ) value was obtained by conducting a Box-Cox transformation analysis using the TRANSREG procedure of SAS. Data subjected to transformation were used to calculate *P*-values. Associations between variables and the outcomes of interest were evaluated using mixed-effects linear models (PROC MIX). Horn bud measurements of male dairy-bred and suckler-bred calves were analysed with the fixed effects of year, breed and age included in the model. Bud measurements of suckler beef calves (CH, LM, SI) (*n* = 191) were analysed and the fixed effects of sex, age and year included in the model. The corresponding non-transformed least squares means (Lsmeans) with standard error of the mean (SEM) are presented to facilitate interpretation of the results. Differences between the Lsmeans were tested using the PDIFF option in SAS or Tukey where appropriate. Lsmeans were considered significantly different at a probability level of *P <* 0.05. Simple linear regression analysis (PROC REG) was used to estimate the equation of the line that best described the association between age with bud size (diameter and height) for each breed and for sex within breed. The regression coefficients and coefficient of determination (R square) were calculated. The PROC CORR procedure Spearman rank correlation (*r*_sp_) was used to describe the correlation matrix on the variables, diameter (mm) with height (mm) data for each breed and for sex within breed. Multivariable linear regression (PROC GLM MANOVA in SAS 9.4) was used to compare the slopes generated from the linear regression analyses.

## Results

### Age at disbudding

In the present study, 16% of HF males calves were disbudded between 14 and 28 d and the remaining 84% between 29 and 60 d with 50% of suckler-bred calves disbudded at either 14–28 d or 29–60 d old. The mean (SD) age of HF male calves at disbudding was 37 [10] days while the corresponding ages for the suckler-bred female and male calves was 26 [8] (CH), 28 [9] (LM), 23 [6] (SI) d, and 24 [7] (CH), 26 [7] (LM) and 23 [6] (SI) d, respectively.

### Effect of breed and age on horn bud diameter and height of dairy-bred and suckler-bred male calves

There was no breed × age interaction (*P >* 0.05) for horn bud diameter or height. There was an effect of breed and of age on horn bud diameter (*P <* 0.0075, *P <* 0.017, respectively) and height (*P <* 0.0086, *P <* 0.0073, respectively) (Table 1). HF calves had a greater (*P <* 0.01) horn bud diameter and height compared to suckler-bred calves, with no difference (*P >* 0.05) among CH, LM or SI sired suckler breeds. Horn bud diameter and height was greater (*P <* 0.05) in calves disbudded at 28–60 d compared to 14–28 d old calves. There was no year × age interaction (*P >* 0.05) and an effect of year where horn bud diameter was greater (*P <* 0.05) in 2019 compared to 2018 with no difference (*P >* 0.05) in height.

**Table 1:** Effect of breed, age and year on horn bud diameter and height of male dairy-bred and suckler-bred calves at disbudding (*n* = 181)

Variable	HF	CH	LIM	SI	SE	14–28 d	29–60 d	SE	2018	2019	SE	Breed	Age	Year	Breed × age	Year × age	Year × Breed × Age
Diameter (mm)	16.97^a^	15.09^b^	13.76^b^	14.41^b^	0.62	14.31	15.81^b^	0.44	14.31	15.80*	0.44	**	*	*	NS	NS	NS
Height (mm)	7.79^a^	5.10^b^	4.69^b^	5.39^b^	0.62	4.88	6.60^b^	0.44	5.91	5.57	0.44	**	**	NS	NS	NS	NS

^a,b^ Within rows, Lsmeans differ between breed, age categories and year (*P <* 0.05)

*HF* Holstein-Friesian, *CH* Charolais, *LM* Limousin, *SI* Simmental

*d* days old at disbudding, *NS* not significant (*P >* 0.05). * *P <* 0.05, ** *P <* 0.01

*SEM* pooled standard error

### Effect of breed and sex on horn bud diameter and height of suckler-bred calves

There was no sex × age interaction or breed × age interaction for horn bud measurements of suckler-bred beef calves (Table 2). There was an effect of sex and age on horn bud diameter and height with suckler-bred M beef calves having greater (*P <* 0.01) horn bud diameter and height than suckler-bred F calves (Table 2). Horn bud diameter and height was greater (*P <* 0.05) on suckler-bred calves disbudded at 28–60 d old compared to 14–28 d calves.

**Table 2:** Effect of sex, age and year on horn bud diameter (mm) and height (mm) of suckler-bred male and female calves at disbudding (*n* = 191)

										***P*** Value					
Variable	Female	Male	SE	14–28 d	29–60 d	SE	2018	2019	SE	Sex	Age	Year	Sex × Age	Sex × Year	Year × Age
Diameter (mm)	13.29^a^	14.46^b^	0.278	13.16	14.58 ^b^	0.274	13.37 ^a^	14.38 ^b^	0.278	**	**	*	NS	NS	NS
Height (mm)	3.88^a^	5.01^b^	0.214	4.11	4.78 ^b^	0.201	4.88 ^a^	4.01^b^	0.214	**	*	**	NS	NS	NS

^a,b^ Within rows, Lsmeans differ between year, sex and age categories (*P <* 0.05)

*d* days old at disbudding, *NS* not significant (*P >* 0.05). * *P <* 0.05, ** *P <* 0.01

*SE* pooled standard error

The slopes of the lines of best fit show that horn bud diameter and height increased with age (*P <* 0.05) for HF, SI male and CH female calves while there was no relationship with age for CH and LM male calves, or for SI and LM female calves (*P >* 0.05). Linear regression of age with diameter and with height for each breed and sex are shown in Table 3. The variability in the data was high as indicated by R-squared values (Table 3). The values ranged from 0.003–0.41 indicating that in the case of the diameter and the height, the weight of the fitting effect was poor. The horn bud growth (slope) of dairy-bred male calves was 0.20 mm/day for diameter and 0.22 mm/day for height while the growth (slope) of suckler-bred beef calves was less than 0.1 mm/day.

**Table 3:** Linear regression analysis describing the line of best fit for the relationship between age with diameter (mm), and age with height (mm) for dairy-bred M calves and suckler-bred M and F calves at disbudding (showing R square and level of significance (*P*-value))

Variable (mm)	Breed and number	Sex	Linear regression	R square	***P***-value
Diameter	HF (n = 88)	M	y = 0.2044x + 10.2470	0.320	< 0.0001
Height	HF (*n* = 88)	M	y = 0.2249x + 0.2481	0.410	< 0.0001
Diameter	LM (*n* = 32)	M	y = 0.0374x + 12.990	0.093	0.0903
Height	LM (*n* = 32)	M	y = 0.0512x + 3.679	0.073	0.1343
Diameter	LM (*n* = 35)	F	y = 0.0742x + 10.613	0.091	0.0786
Height	LM (*n* = 35)	F	y = − 0.0512x + 3.679	0.073	0.1343
Diameter	CH (*n* = 39)	M	y = 0.0507x + 13.932	0.073	0.0872
Height	CH (n = 39)	M	y = 0.0507x + 13.932	0.073	0.0872
Diameter	CH (*n* = 47)	F	y = 0.0752x + 11.73	0.175	0.0031
Height	CH (n = 47)	F	y = 0.0049x + 3.5037	0.003	0.7209
Diameter	SI (*n* = 22)	M	y = 0.0606x + 13.02	0.279	0.0097
Height	SI (*n* = 22)	M	y = 0.0606x + 13.02	0.279	0.0097
Diameter	SI (*n* = 16)	F	y = − 0.00091x + 12.392	0.088	0.2482
Height	SI (*n* = 16)	F	y = − 0.0413x + 3.8698	0.036	0.4672

*HF* Holstein-Friesian, *CH* Charolais, *LM* Limousin, *SI* Simmental

*d* days old at disbudding, *M* male, *F* female

The correlation coefficients between diameter with height measurements were correlated for all breeds (*P <* 0.05) and for sex (*P <* 0.05) within breed (Table 4).

**Table 4:** Correlation matrix of diameter with height for dairy-bred and suckler-bred M and F calves at disbudding, coefficient of determination (R square) and level of significance (*P*-value)

Variable (mm)	Breed and number	Sex	Intercept	R square	***P*** value
Diameter	HF (n = 88)	M	−3.5055	0.483	< 0.0001
Height	HF (n = 88)	M			
Diameter	LM (n = 32)	M	−4.541	0.593	< 0.0001
Height	LM (n = 32)	M			
Diameter	LM (n = 35)	F	−2.7102	0.446	< 0.0001
Height	LM (n = 35)	F			
Diameter	CH (n = 39)	M	9.79865	0.652	< 0.0001
Height	CH (n = 39)	M			
Diameter	CH (n = 47)	F	−0.68857	0.268	0.0002
Height	CH (n = 47)	F			
Diameter	SI (n = 22)	M	−2.7102	0.446	< 0.0001
Height	SI (n = 22)	M			
Diameter	SI (n = 16)	F	−3.1919	0.550	0.0007
Height	SI (n = 16)	F			

*HF* Holstein-Friesian, *CH* Charolais, *LM* Limousin, *SI* Simmental. *d* days old at disbudding. *M* male. *F* female. Correlation (effect size and the strength of the correlation) was described using the following: 0.00–0.19 “very weak”; 0.20–0.39 “weak”; 0.40–0.59 “moderate”; 0.60–0.79 “strong”; 0.80–1.0 “very strong”

Multivariable linear regression models (GLIMMIX procedure) was used to compare age with horn bud measurements for HF and suckler-bred (CH, LM, SM) male calves (Table 5). The diameter and height was 3.54 mm and 3.00 mm, respectively, greater than the intercepts (15.87 mm, 6.18 mm respectively) for HF male calves compared with the suckler-bred beef calves while there was no difference among the suckler-bred M calves. Calves disbudded at 29–60 d had greater horn bud diameter (increase of 1.31 mm) and height (increase of 1.84 mm) than calves disbudded at 14–28 d old (*P <* 0.01) (Table 5).

**Table 5:** Multivariable linear regression of breed, age and year on horn bud diameter and height of dairy-bred and suckler-bred male calves at disbudding (*n* = 181)

	Estimate	SE	P value	Breed	Age	Year
**Horn bud diameter**						
Intercept	15.87	0.715	<.0001	<.0001	0.011	<.0001
HF	3.54	0.733	<.0001			
CH	1.05	0.750	0.1617			
LM	0.07	0.784	0.9243			
SI	0.00^a^	.	.			
Age 14–28 d	0.00^b^	.	.			
Age 29–60 d	1.31	0.509	0.011			
2018	0.00^c^	.	.			
2019	2.49	0.427	<.0001			
**Horn bud height**						
Intercept	6.18	0.709	<.0001	<.0001	0.0003	0.4509
HF	3.00	0.727	<.0001			
CH	0.25	0.743	0.7336			
LM	0.13	0.777	0.8689			
SI	0.00^a^	.	.			
Age 14–28 d	0.00^b^	.	.			
Age 29–60 d	1.84	0.504	0.0003			
2018	0.00^c^	.	.			
2019	0.32	0.424	0.4509			

*HF* Holstein-Friesian, *CH* Charolais, *LM* Limousin, *SI* Simmental

*d* days old at disbudding. ^a^ Referent for breed. ^b^Referent for age. ^c^Referent for year

Multivariable linear regression models (GLIMMIX procedure) was used to compare the regression lines for effect of suckler breed (CH, LM, SM), sex and age (Table 6) on horn bud size. The diameter and height for the suckler-bred CH, LM, SM was not different from the intercepts for diameter (15.09 mm) and height (4.71 mm). There was a sex effect for diameter and height where male calves had a diameter of 1.33 mm and a height of 1.01 mm greater than the corresponding intercepts (15.09 mm, 4.71 mm, respectively) when compared to suckler-bred F calves. Calves disbudded at 29–60 d had greater horn bud diameter (increase of 1.32 mm) (*P <* 0.05) and height (increase of 0.64 mm) (*P <* 0.05) than calves disbudded at 14–28 d old.

**Table 6:** Multivariable linear regression of age, sex and year on horn bud diameter and height of suckler-bred male and female calves at disbudding (*n* = 191)

	Estimate	SE	***P*** value	Breed	Sex	Age	Year
**Horn bud diameter**							
Intercept	**15.09**	0.563	<.0001	0.0576	0.0003	0.0009	0.0823
CH	0.96	0.493	0.0533				
LM	0.05	0.515	0.9155				
SIM	0.00^a^	.	.				
Female	0.00^b^	.	.				
Male	1.33	0.360	0.0003				
Age 14–28 d	0.00^c^	.	.				
Age 29–60 d	1.32	0.391	0.0009				
2018	0.00^d^	.	.				
2019	0.68	0.389	0.0823				
**Horn bud height**							
Intercept	**4.71**	0.443	<.0001	0.719	0.0004	0.0536	0.0055
CH	0.24	0.388	0.5358				
LM	0.01	0.405	0.9818				
SIM	0.00^a^	.	.				
Female	0.00^b^	.	.				
Male	1.01	0.284	0.0004				
Age 14–28 d	0.00^c^	.	.				
Age 29–60 d	0.60	0.308	0.0536				
2018	0.00^d^	.	.				
2019	0.86	0.306	0.0055				

*CH* Charolais, *LM* Limousin, *SI* Simmental

*d* days old at disbudding. ^a^ Referent for breed. ^b^Referent for sex. ^c^Referent for age. ^d^Referent for year.

## Discussion

To the best of our knowledge, this is the first study that has quantified horn bud size of dairy-bred and suckler-bred calves at time of disbudding. In the present study, the dairy-bred calves were purchased after 28 d old directly from farms and assembled at Teagasc Grange. The dairy-bred calves were disbudded within 1 week after arrival and a greater number were disbudded at an older age in 2019.

There is limited information in the literature on age at cautery disbudding for suckler-bred beef calves with most studies reporting on cautery disbudding of dairy-bred calves. For example, the mean disbudding age in the present study was greater than that reported by Stanek et al. [17] (median 20 d; 2.9 week) for dairy calves on Czech farms, but lower than 6.4 weeks (median) in Canada [15] and 4.6 weeks (mean) in Italy [6]. The age range at disbudding reported by Stanek et al. [17] on surveyed Czech farms ranged from 3 to 68 d of age. A possible reason why calves were disbudded before 28 d of age on Czech farms is that legislation allows the disbudding of calves without the use of pain relief up to 28 d. In the Stanek et al. [17] study, 63.3% of calves were disbudded before 28 d of age, which is greater than those reported in other studies: 21% in Canada [28], 25.7% in the US [29] and 17% in southern Brazil [20] and lower than those reported (95%) in Finland [30].

In the present study, horn bud diameter and height were very variable across breeds which suggest that most of the variation is not explained by age. It is possible that some of the variation, at least for the suckler-bred calves, may be attributed to calf sex since male suckler-bred calves had a greater horn bud height (29.1%) and diameter (8.80%) compared with female suckler calves at time of disbudding. However, this finding is unsurprising for male calves as there is general agreement that the main evolutionary benefit of males having larger horns than females relates to intra-sexual competition for mates [7, 31, 32]. In the present study, while no dairy-bred females were included in the study, it would be of interest to quantify the horn bud measurements of male and female dairy-bred calves at time of disbudding.

A number of studies have focused on the acute pain response to cautery disbudding [33–36] and of the benefits of providing local anaesthesia and pain relief [12, 37–41] at the time of disbudding. Adcock and Tucker [36] reported that calves disbudded at 3 d versus 35 d of age were more sensitive to pain after disbudding. Similar findings of increased sensitivity are reported by Casoni et al. [42] for up to 14 weeks post-disbudding of 7 d and 28 d old Holstein calves. However, while local anaesthesia in addition to analgesia are of benefit in mitigating the pain related disbudding behaviours, few studies have investigated the responses of contrasting breeds, sex and age of calves on recommendations for age-related anaesthetic and analgesic protocols. Caray et al. [43] reported some effects of age, breed or sex on the behavior of calves that were observed between 2 and 7 h after disbudding (for example 4-week calves and Charolais calves were more active than their 1-week and Holstein counterparts), but found no interactions between age, breed, sex and treatment medication.

The degree of tissue damage associated with disbudding is reported to be influenced by the stage of development of the horn bud (36). For example, in younger calves the burning of the vessels surrounding the horn bud is sufficient, whereas the whole bud needs to be removed (by levering it out from the side) when the horn is further developed [36]. Therefore, depending on the procedure, calves with greater horn bud diameter at time of disbudding may have more tissue damage which could lead to prolonged healing time and prolonged stress. Disbudding of smaller horn buds may be preferential since less tissue is damaged and it could decrease wound healing time.

In the present study, the horn bud size (diameter and height) was greater for dairy-bred than suckler-bred male calves at time of disbudding, and male suckler-bred calves had greater diameters and heights compared to suckler-bred female calves. Additionally, the disbudding of calves with an appropriate disbudder tip size is a consideration and may be a more practical strategy for mitigating pain and improving wound healing. Empirical evidence is lacking on the stress response and wound healing time of calves, when horn buds of varying size are removed using an appropriate disbudder tip diameter. At the time of disbudding, the horn bud should be large enough to be visible/palpable, but not so large that the disbudding device doesn’t work or that the trauma is greater. Similarly, the calf should be old enough to have a horn bud but not so old that their horns are too large as this would greatly increase trauma and prolong wound healing time.

## Conclusions

Setting definite ages for disbudding is difficult. The horn bud size (diameter and height) was greater in dairy-bred calves than suckler-bred beef calves at time of disbudding, and male suckler-bred calves had greater horn bud size than female counterparts. This information would be useful to guide more specific recommendations on calf disbudding. The association between age and horn bud size was very weak in dairy-bred and suckler-bred calves. Further work needs to investigate effect of disbudding horn buds of different size on pain responses, behaviour, horn re-growth, and post-disbudding trauma/infections in particular types or breeds of cattle before a more concrete recommendation can be made.

## Data Availability

All data supporting these research findings are included within the manuscript. The databases (without personally identifiable information) are available from the corresponding author upon request.

## Abbreviations

M: Male
F: Female
d: day
mo: month
HF: Holstein-Friesian
CH: Charolais
LM: Limousin
SI: Simmental
